# High-level production of membrane proteins in *E. coli* BL21(DE3) by omitting the inducer IPTG

**DOI:** 10.1186/s12934-015-0328-z

**Published:** 2015-09-16

**Authors:** Zhe Zhang, Grietje Kuipers, Łukasz Niemiec, Thomas Baumgarten, Dirk Jan Slotboom, Jan-Willem de Gier, Anna Hjelm

**Affiliations:** Department of Biochemistry and Biophysics, Center for Biomembrane Research, Stockholm University, 106 91 Stockholm, Sweden; Xbrane Bioscience AB, 111 45 Stockholm, Sweden; Groningen Biomolecular Sciences and Biotechnology Institute, University of Groningen, Groningen, The Netherlands

**Keywords:** *Escherichia coli*, Protein production, Membrane protein, Secretory protein, BL21(DE3), T7 RNA polymerase

## Abstract

**Background:**

For membrane protein production, the *Escherichia coli* T7 RNA polymerase (T7 RNAP)-based protein production strain BL21(DE3) in combination with T7-promoter based expression vectors is widely used. Cells are routinely cultured in Lysogeny broth (LB medium) and expression of the chromosomally localized *t7rnap* gene is governed by the isopropyl-β-d-1-thiogalactopyranoside (IPTG) inducible *lac*UV5 promoter. The T7 RNAP drives the expression of the plasmid borne gene encoding the recombinant membrane protein. Production of membrane proteins in the cytoplasmic membrane rather than in inclusion bodies in a misfolded state is usually preferred, but often hampered due to saturation of the capacity of the Sec-translocon, resulting in low yields.

**Results:**

Contrary to expectation we observed that omission of IPTG from BL21(DE3) cells cultured in LB medium can lead to significantly higher membrane protein production yields than when IPTG is added. In the complete absence of IPTG cultures stably produce membrane proteins in the cytoplasmic membrane, whereas upon the addition of IPTG membrane proteins aggregate in the cytoplasm and non-producing clones are selected for. Furthermore, in the absence of IPTG, membrane proteins are produced at a lower rate than in the presence of IPTG. These observations indicate that in the absence of IPTG the Sec-translocon capacity is not/hardly saturated, leading to enhanced membrane protein production yields in the cytoplasmic membrane. Importantly, for more than half of the targets tested the yields obtained using un-induced BL21(DE3) cells were higher than the yields obtained in the widely used membrane protein production strains C41(DE3) and C43(DE3). Since most secretory proteins reach the periplasm via the Sec-translocon, we also monitored the production of three secretory recombinant proteins in the periplasm of BL21(DE3) cells in the presence and absence of IPTG. For all three targets tested omitting IPTG led to the highest production levels in the periplasm.

**Conclusions:**

Omission of IPTG from BL21(DE3) cells cultured in LB medium provides a very cost- and time effective alternative for the production of membrane and secretory proteins. Therefore, we recommend that this condition is incorporated in membrane- and secretory protein production screens.

**Electronic supplementary material:**

The online version of this article (doi:10.1186/s12934-015-0328-z) contains supplementary material, which is available to authorized users.

## Background

The *Escherichia coli* T7 RNA polymerase-based protein production strain BL21(DE3) in combination with T7 promoter-based expression vectors is widely used to produce recombinant proteins [1–3]. In BL21(DE3), expression of the gene encoding the recombinant protein is transcribed by the chromosomally encoded T7 RNA polymerase (T7 RNAP), which transcribes eight times faster than *E. coli* RNAP [4–6]. The gene encoding the T7 RNAP is under control of the *lac*UV5 promoter (P_*lac*UV5_), which is a strong variant of the wild-type *lac* promoter [7–9]. Addition of isopropyl-β-d-1-thiogalactopyranoside (IPTG) leads to expression of the gene encoding the T7 RNAP. The T7 RNAP specifically recognizes the T7 promoter, which drives the expression of the gene encoding the recombinant protein [4, 5]. The rationale behind BL21(DE3) is very simple: the higher the mRNA levels, the more recombinant protein can be produced. Notably, P_*lac*UV5_ is in BL21(DE3) a poorly-titratable promoter. Expression of genes encoding recombinant proteins, in particular those encoding membrane proteins, can be toxic to BL21(DE3) [10]. The toxicity of membrane protein production appears to be mainly caused by saturation of the capacity of the Sec-translocon, which is a protein-conducting channel in the cytoplasmic membrane assisting the biogenesis of membrane proteins and translocation of secretory proteins across this membrane [11]. Saturating the Sec-translocon capacity negatively affects both biomass formation and membrane protein production yields [12, 13]. It should be noted that it is preferred to produce membrane proteins in a membrane system rather than in inclusion bodies, since it greatly facilitates the isolation of membrane proteins for structural and functional studies [14].

To deal with the toxic effects that the production of recombinant proteins can cause, variants of BL21(DE3) harbouring plasmids with the gene encoding the T7 lysozyme can be used [15]. The T7 lysozyme is a natural inhibitor of the T7 RNAP and by governing the expression of *t7lys* using different promoter systems the activity of T7 RNAP can be modulated, which leads to lower recombinant protein production rates. This can reduce the toxic effects caused by recombinant protein production, thereby enhancing yields. However, the *t7lys* expression plasmids require the use of an additional antibiotic and sometimes also an inducer for regulating *t7lys* expression, thereby adding another layer of complexity [13]. Another strategy to overcome the toxic effects caused by the production of recombinant proteins is to screen for mutant strains with improved protein production characteristics [16, 17]. Prime examples of such mutant strains are the BL21(DE3)-derived strains C41(DE3) and C43(DE3), also referred to as the Walker strains [16]. These strains are now widely used to produce proteins, in particular membrane proteins [2]. Recently, we have shown that mutations weakening P_*lac*UV5_ governing expression of *t7rnap* are key to the improved membrane protein production characteristics of the Walker strains and are actually selected for upon the production of any protein in BL21(DE3) [13, 18]. The mutations weakening P_*lac*UV5_ result in the production of much lower amounts of T7 RNAP upon induction of expression of *t7rnap* with IPTG than in BL21(DE3). As a consequence the membrane protein production rates are lowered, thereby averting saturation of the Sec-translocon capacity. This leads to improved membrane protein production yields in the cytoplasmic membrane.

While we were in the process of screening for improved production of the *E. coli* integral membrane chaperone YidC and the *E. coli* glutamate proton symporter GltP in BL21(DE3) cells cultured in lysogeny broth (LB medium), we made an unexpected observation. We observed that these two membrane proteins could be efficiently produced without adding any IPTG. Literature searches showed that it had been observed before that BL21(DE3) cells cultured in LB medium can produce proteins in the absence of IPTG and that the mechanism driving the induction of *t7rnap* expression in the absence of IPTG is not clear [19, 20]. To our surprise, membrane protein production in BL21(DE3) in the absence of IPTG had never been studied in a more systematic and comparative manner. Here, we show that culturing BL21(DE3) cells in LB medium in the absence of the inducer IPTG provides a cost-effective, simple and competitive alternative for the production of membrane- as well as secretory proteins.

## Results and discussion

### Omitting the inducer IPTG from BL21(DE3) cells cultured in LB medium leads to enhanced production of the membrane proteins YidC and GltP

**Fig. 1:**
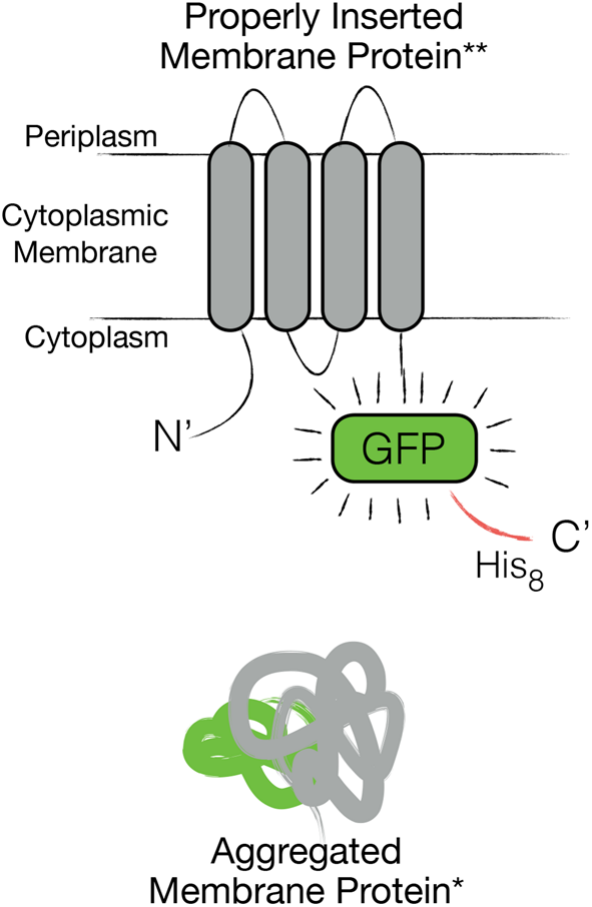
Membrane protein GFP fusions. In this study, membrane proteins were produced as C-terminal GFP fusions. The GFP moiety only folds properly and becomes fluorescent when the membrane protein-GFP fusion is inserted in the cytoplasmic membrane. When the membrane protein GFP fusion aggregates in the cytoplasm the GFP moiety does not fold properly and does not fluoresce

**Fig. 2:**
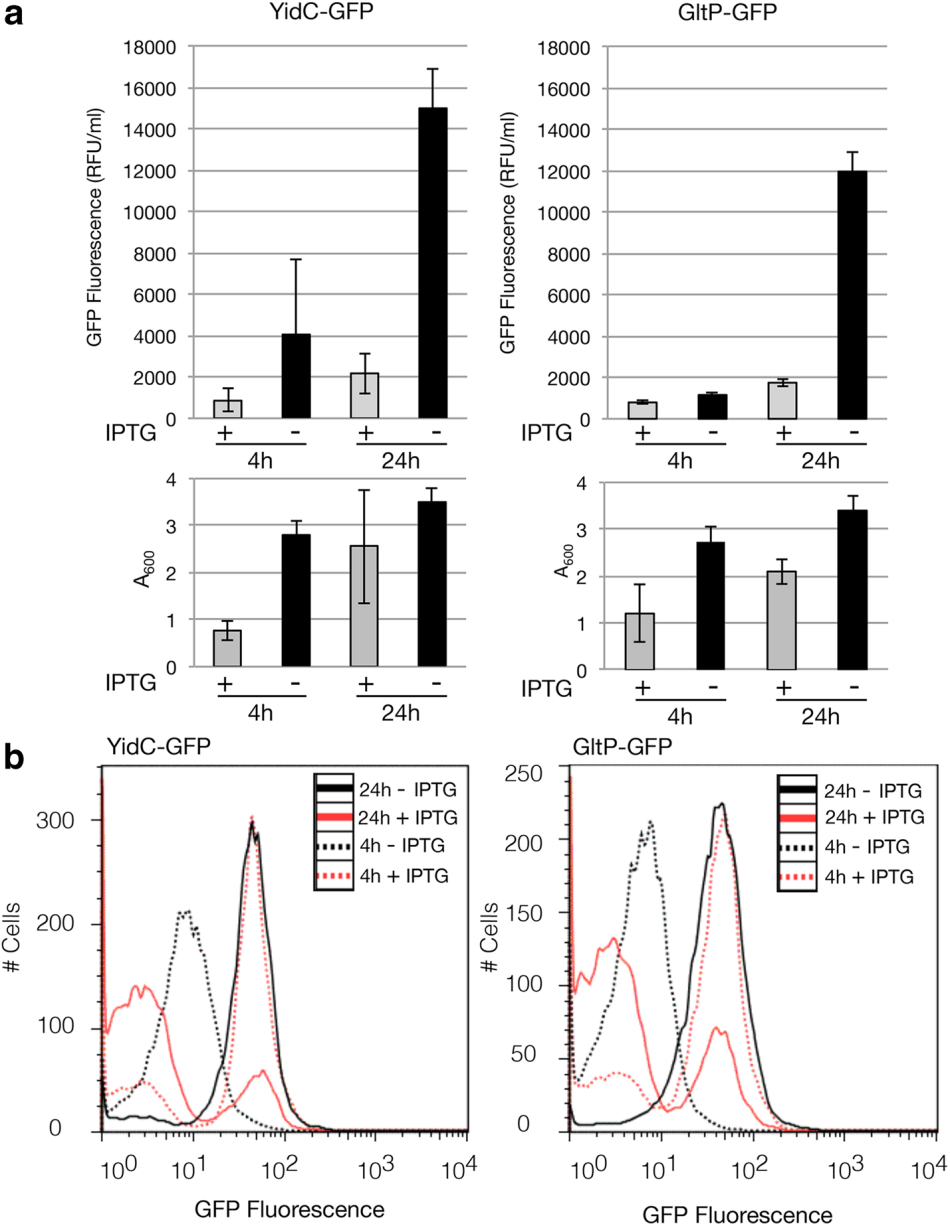
Production of YidC-GFP and GltP-GFP in BL21(DE3) cells in the presence and absence of IPTG. BL21(DE3) cells harboring either a pET-based *yidC*-*gfp* or *gltP*-*gfp* expression vector were cultured in LB medium at 30 °C in the presence and absence of IPTG (final concentration 0.4 mM). Membrane protein production and biomass formation were monitored 4 and 24 h after the addition of IPTG. **a** YidC-GFP and GltP-GFP production levels in the cytoplasmic membrane of BL21(DE3) cells cultured in the presence and absence of IPTG were assessed by monitoring fluorescence (relative fluorescence unit, RFU) per milliliter of culture. Biomass formation was monitored by measuring the A_600_. RFUs/ml per A_600_ are shown in Additional file 1: Figure S1. **b** The production of membrane protein-GFP fusion per cell was determined using flow cytometry. Traces of cells cultured in the prescence of IPTG are in red and traces of cells cultured in the absence of IPTG are in *black*. Cells harvested after 4 h are represented by *dotted lines* and cells harvested after 24 h are represented by *solid lines*

We routinely use the integral membrane chaperone YidC and the glutamate proton symporter GltP as model membrane proteins to develop cost- and time-effective membrane protein production strategies (e.g., [21]). To facilitate the detection of produced membrane proteins in the cytoplasmic membrane, all target membrane proteins are C-terminally fused to GFP (Fig. 1) [22]. While we were in the process of screening the production of YidC and GltP in BL21(DE3) cells cultured in LB medium, we included as negative controls cultures of BL21(DE3) to which the inducer IPTG was not added. Fluorescence of IPTG induced cultures was monitored 4 and 24 h after the addition of IPTG (Fig. 2a). At the same time-points the fluorescence of the non-IPTG induced cultures was also measured (Fig. 2a). To our surprise, after 24 h, the fluorescence intensities per ml of un-induced cultures were more than five times higher than that of IPTG induced cultures. Also, the A_600_ values of these cultures were higher than the ones of the IPTG induced cultures (Fig. 2a, Additional file 1: Figure S1).

In all cultures, we monitored GFP fluorescence in individual cells using flow cytometry (Fig. 2b). In the absence of IPTG, cultures producing YidC-GFP and GltP-GFP consisted of a homogenous population of cells, both after 4 and 24 h, and the fluorescence per cell increased over time (Fig. 2b). However, when cells were cultured in the presence of IPTG the cultures consisted of a mixture of producing and non-producing cells, both after 4 and 24 h [21]. The increase of the fraction of non-producing cells over time in IPTG induced cultures indicates that non-producing cells are selected for in the presence of IPTG. This explains why the biomass formation in IPTG induced cultures appears to catch up after 24 h (Fig. 2a). In the presence of IPTG, the fluorescence per cell in the producing population after 4 and 24 h was similar to the fluorescence per cell in the absence of IPTG after 24 h. However, the dramatic increase of the fraction of non-producing cells in the presence of IPTG along with the lower biomass formation results in lower overall production yields.

Thus, when IPTG is omitted from BL21(DE3)/LB medium-based cultures, both YidC-GFP and GltP-GFP appear to be more efficiently produced than when IPTG is added to the cultures.

### Characterizing YidC-GFP and GltP-GFP production

**Fig. 3:**
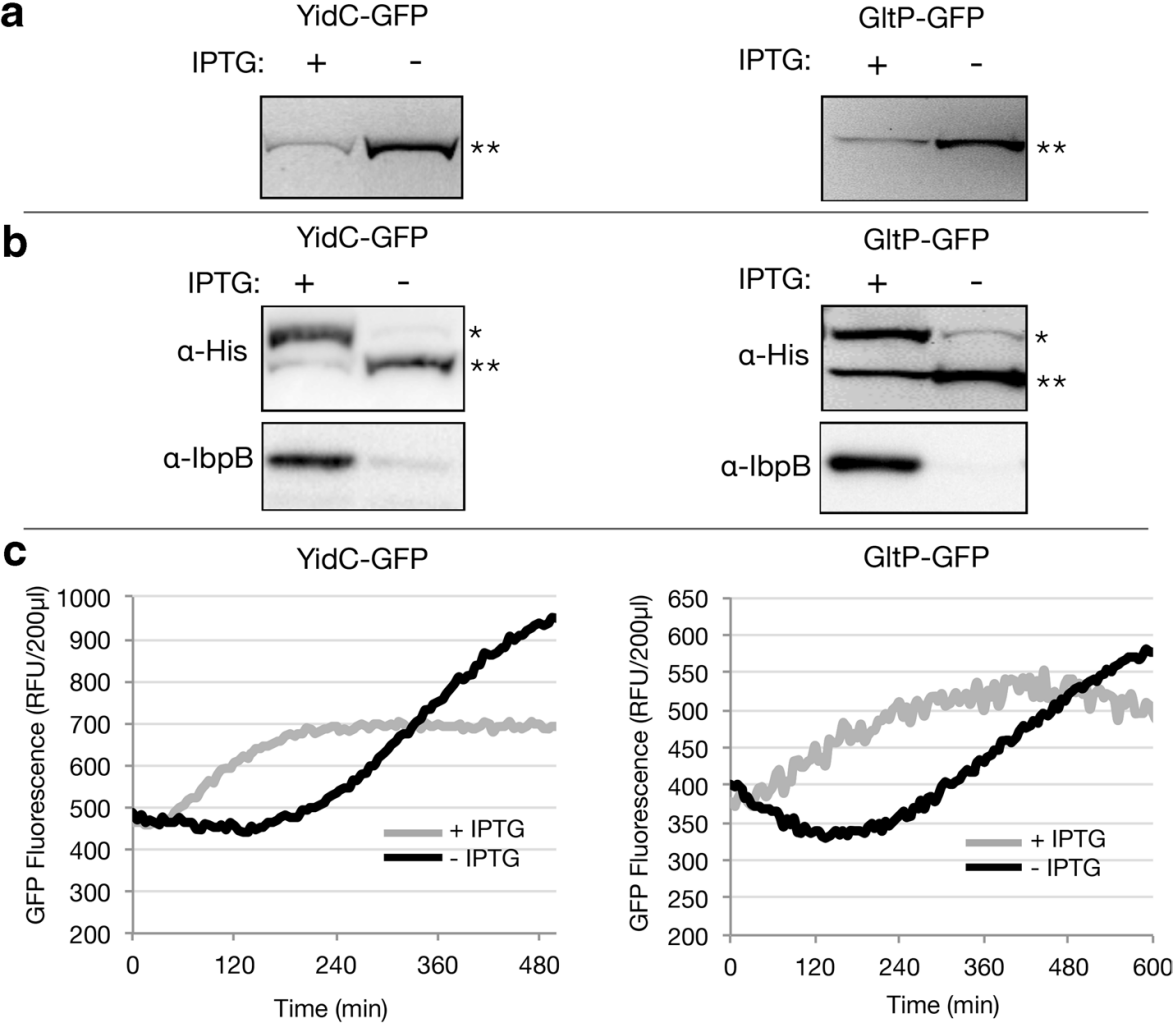
Characterizing YidC-GFP and GltP-GFP production. BL21(DE3) cells harboring either a pET-based *yidC*-*gfp* or *gltP*-*gfp* expression vector were cultured in LB medium at 30 °C in the absence and presence of IPTG (final concentration 0.4 mM). **a** The integrity of the in the cytoplasmic membrane produced YidC-GFP and GltP-GFP fusions (*double asterisk*) was monitored in whole-cell lysates using in-gel fluorescence 24 h after the addition of IPTG. 0.05 A_600_ units of cells were loaded per lane. **b** The ratio of the cytoplasmic membrane inserted to non-inserted YidC-GFP and GltP-GFP was monitored 24 h after the addition of IPTG. Levels of non-inserted (*asterisk*; see also Fig. 1) and inserted (*double asterisk*; see also Fig. 1) membrane protein-GFP fusions in whole-cell lysates were analyzed by means of SDS-PAGE followed by immuno-blotting using an antibody recognizing the His-tag at the C-terminus of the GFP moiety (*top panels*). Note that the inserted membrane protein-GFP fusions correspond with the fluorescent bands detected using in-gel fluorescence; both are marked with *double asterisk*. Protein folding/aggregation stress in the cytoplasm was monitored by determining the levels of IbpB in whole-cell lysates using immuno-blotting (*bottom panels*). 0.05 A_600_ units of cells were loaded per lane. **c** The production of YidC-GFP and GltP-GFP in the cytoplasmic membrane was monitored on-line by measuring GFP fluorescence every 5 min in cells cultured in the presence and absence of IPTG in a 96-well plate in a spectrofluorometer. Cells cultured in the presence of IPTG are represented in *grey* and cells cultured in the absence of IPTG are represented in *black*

To characterize the YidC-GFP and GltP-GFP production process in more detail, we first monitored the integrity of YidC-GFP and GltP-GFP, produced in the cytoplasmic membrane, using in-gel fluorescence [22]. Proteins from whole-cell lysates were separated by SDS-PAGE and subsequently the gel was illuminated with UV light and GFP fluorescence in the gel was captured using a CCD camera (Fig. 3a). For both YidC-GFP and GltP-GFP only one fluorescent band could be detected and they both had the expected molecular weight. The fluorescent bands in lysates of cells cultured in the absence of IPTG were more intense than the ones of cells cultured in the presence of IPTG, which is in keeping with the whole cell fluorescence measurements.

Next, we used an SDS-PAGE/immuno-blotting-based assay that can distinguish between membrane integrated and non-integrated material (see also Fig. 1) [23]. The assay in short: if a membrane protein-GFP fusion is not inserted in the cytoplasmic membrane and ends up in aggregates, its GFP moiety does not fold properly. The GFP moiety folds properly and becomes fluorescent only if the membrane protein-GFP fusion is inserted in the cytoplasmic membrane. Correctly folded GFP is not denatured in SDS-PAGE solubilisation buffer at temperatures below 37 °C. As a consequence, a membrane protein-GFP fusion that has been inserted in the cytoplasmic membrane will migrate faster in a gel than a non-inserted fusion. We monitored the behaviour of both YidC-GFP and GltP-GFP produced in BL21(DE3) in the presence and absence of IPTG using this assay. Through immuno-blotting with an antibody directed against a His-tag, C-terminally attached to GFP, both YidC-GFP and GltP-GFP were detected. In lysates from cells cultured in the presence of IPTG both YidC-GFP and GltP-GFP showed up as two bands: a weak fluorescent one (cytoplasmic membrane integrated) and an intense non-fluorescent one with a higher apparent molecular weight, representing aggregated material in the cytoplasm (Fig. 3b). Most of the produced YidC-GFP and GltP-GFP appeared to end up in aggregates when IPTG was added. When IPTG was omitted from the culture, hardly any non-fluorescent YidC-GFP or GltP-GFP was detected (Fig. 3b). We also monitored the levels of inclusion body protein IbpB, which is a sensitive indicator for the accumulation of aggregated proteins in the cytoplasm [24]. IbpB was clearly present in IPTG induced BL21(DE3) cells and was hardly detectable in non-IPTG induced BL21(DE3) cells (Fig. 3b). These observations are consistent with the hypothesis that non-fluorescent membrane protein GFP fusions accumulate in the cytoplasm upon the addition of IPTG [12]. This observation indicates that upon the addition of IPTG, the production of both YidC-GFP and GltP-GFP leads to saturation of the Sec-translocon capacity. In contrast, in the absence of IPTG the Sec-translocon capacity does not appear to be saturated, resulting in higher yields of membrane proteins produced in the cytoplasmic membrane.

There is a correlation between the rate of membrane protein production and saturation of the Sec-translocon capacity [13]. Therefore, we monitored YidC-GFP and GltP-GFP production over time in BL21(DE3) cells cultured in the presence and absence of IPTG [13] (Fig. 3c). The initial membrane protein production rate in cells cultured in the absence of IPTG was lower than in the presence of IPTG. However, over time more GFP fluorescence, i.e., higher levels of membrane inserted target membrane protein, accumulated in cells cultured in the absence of IPTG than in the presence of IPTG. This result is in keeping with the idea that not adding IPTG leads to a membrane protein production regime that does not saturate the Sec-translocon capacity.

**Fig. 4:**
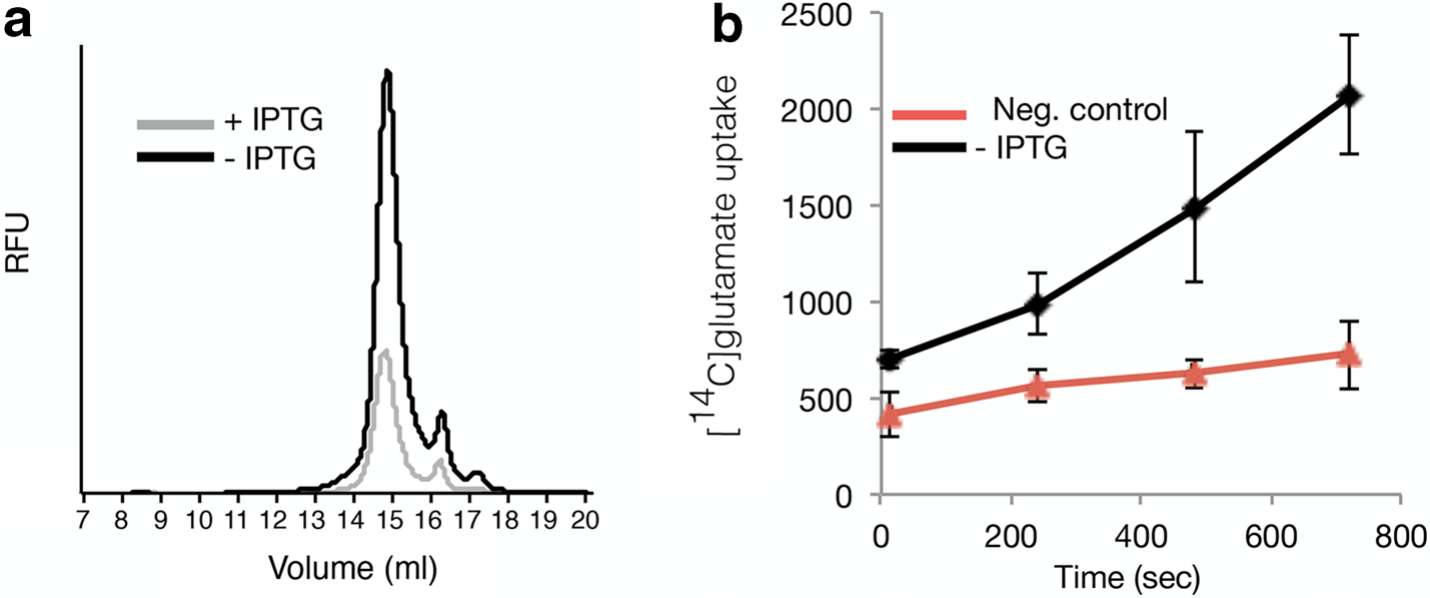
Characterizing in the cytoplasmic membrane produced YidC-GFP and GltP-GFP. BL21(DE3)pET*yidC*-*gfp* and BL21(DE3)pET*gltP*-*gfp* cells cultured in the absence and presence of IPTG as described in the legend of Fig. 3 were harvested and membranes were isolated. **a** The quality of produced YidC-GFP fraction that was inserted into the cytoplasmic membrane was judged by the FSEC profiles of DDM-solubilised membranes. The FSEC trace of YidC-GFP purified from cells cultured in the presence of IPTG is in* grey* (90.3 µg of total protein was loaded containing 0.29 μg of YidC-GFP) and the FSEC trace of YidC-GFP purified from cells cultured in the absence of IPTG is represented in *black* (25.4 µg of total protein was loaded containing 0.43 μg of YidC-GFP) (relative fluorescence unit, RFU). Traces were normalized according to the dilution factor used to obtain equivalent fluorescence intensities prior to solubilisation of the membranes (see “Methods”). **b** GltP-GFP was purified from the membranes and incorporated in liposomes, and glutamate uptake was determined. As a control, liposomes without reconstituted protein were used. Activity measurements of GltP-GFP purified from cells cultured in the absence of IPTG are represented in* black* and activity measurements in plain liposomes are represented in* red*. Note that the amount of GltP-GFP produced in BL21(DE3) cells cultured in the presence of IPTG was insufficient to determine activity

Finally, the produced YidC-GFP and GltP-GFP were characterized in more detail. Cytoplasmic membranes of one liter cultures producing YidC-GFP in the presence and absence of IPTG were isolated. The IPTG induced culture contained 1.3 mg of YidC-GFP per liter and the non-induced culture contained 8.7 mg of YidC-GFP per liter [22]. The total membrane fractions isolated from the IPTG induced culture and from the non-induced culture contained 0.4 and 1.7 mg of YidC-GFP, respectively [22]. Subsequently, the membranes were solubilised in the detergent n-Dodecyl β-d-Maltopyranoside (DDM) and the dispersity of solubilised YidC-GFP was monitored using fluorescence-detection size-exclusion chromatography (FSEC) (Fig. 4a) [25]. YidC-GFP produced in cells both in the presence and absence of IPTG was monodisperse. However, in the absence of IPTG significantly more material was produced. Also cytoplasmic membranes from one liter BL21(DE3)-based cultures producing GltP-GFP in the presence and absence of IPTG were isolated. GltP-GFP was purified and reconstituted in liposomes so that GltP activity (i.e., glutamate uptake) could be monitored. Only membranes isolated from BL21(DE3) cells producing GltP-GFP in the absence of IPTG gave enough material after Immobilized-Metal Affinity Chromatography (IMAC)-based purification to reconstitute GltP-GFP in liposomes and to show that it was active (Fig. 4b) [22]. From a one liter non-induced culture 1.0 mg of GltP-GFP was isolated.

Taken together, omitting IPTG from BL21(DE3)-based cultures greatly increases yields of YidC-GFP and GltP-GFP produced in the cytoplasmic membrane. The produced proteins are of high quality and can be used for further characterization.

### Benchmarking the production of membrane proteins in BL21(DE3) cells cultured in LB medium in the absence of IPTG

**Fig. 5:**
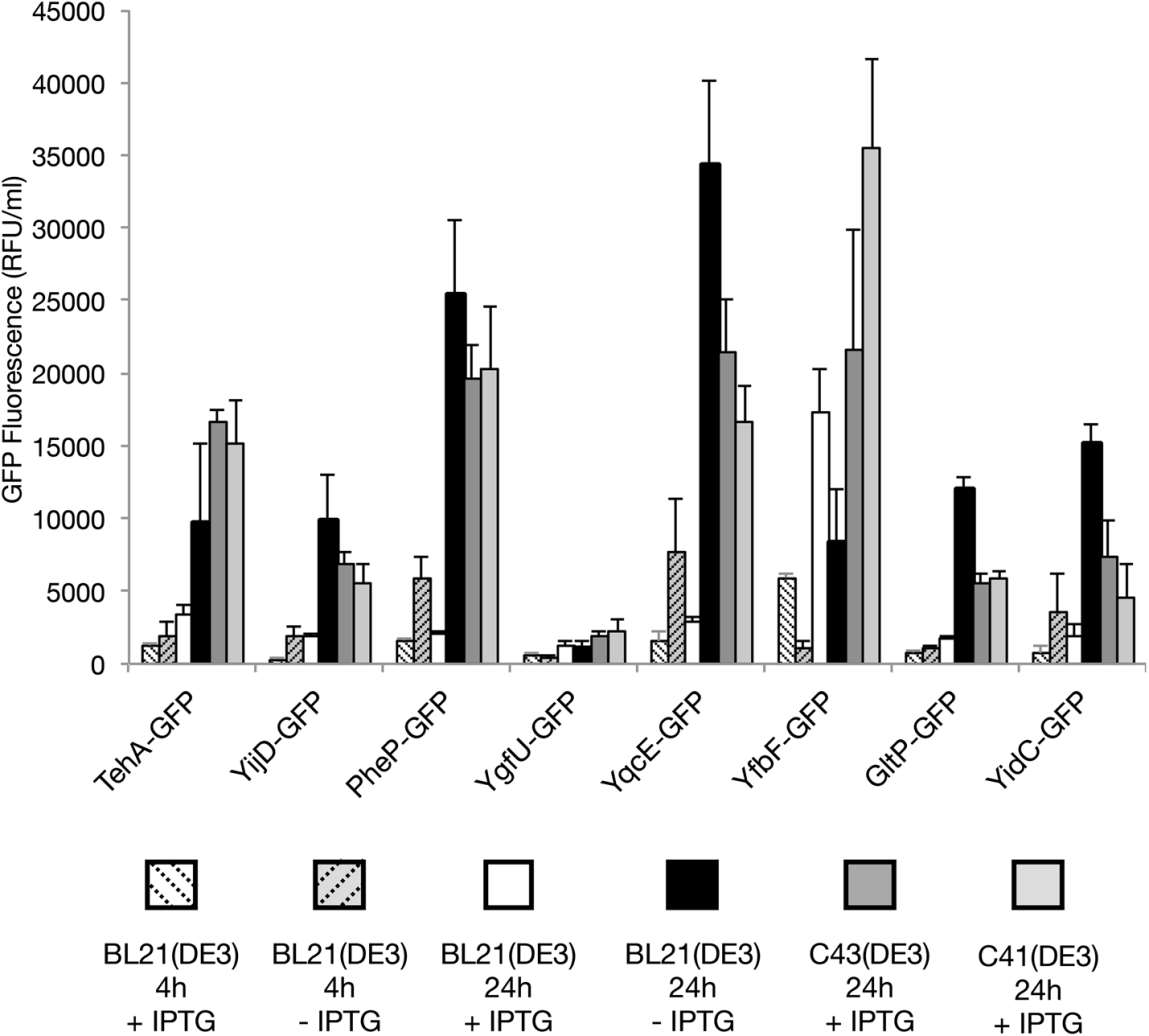
Screening the production of membrane proteins in BL21(DE3), C41(DE3) and C43(DE3). The production of a set of membrane protein GFP-fusions (Additional file 1: Table S1) was assessed in BL21(DE3) cells cultured in the presence and absence of IPTG, and C41(DE3) and C43(DE3) cells cultured in the presence of IPTG. For BL21(DE3)-based cultures membrane protein-GFP production was monitored by measuring GFP fluorescence per ml of culture 4 and 24 h after the addition of IPTG (relative fluorescence unit, RFU). For C41(DE3) and C43(DE3)-based cultures fluorescence per ml of culture was monitored 24 h after the addition of IPTG. RFUs/ml per A_600_ are shown in Additional file 1: Figure S1. Notably, not adding IPTG to C41(DE3) and C43(DE3) cultures leads to lower production levels than adding IPTG (see Additional file 1: Figure S2)

To benchmark the production of membrane proteins in BL21(DE3) cells cultured in LB medium in the absence of IPTG, we used in addition to YidC-GFP and GltP-GFP six more targets and monitored production of all eight membrane proteins also in the C41(DE3) and C43(DE3) strains (Fig. 5) (Additional file 1: Table S1). Both C41(DE3) and C43(DE3) are widely used to produce membrane proteins [10]. The six additional targets were randomly picked and also fused to GFP at their C-termini. For six out of the eight targets tested, production yields obtained for un-induced BL21(DE3)-based cultures were higher than those obtained for IPTG induced cultures. Importantly, for five out of the eight targets tested BL21(DE3)-based cultures to which no IPTG had been added even outperformed C41(DE3) and C43(DE3).

Taken together, membrane protein production yields using BL21(DE3) cells cultured in LB medium without IPTG are in many instances significantly higher than yields obtained with the established membrane protein production strains C41(DE3) and C43(DE3).

### Efficient production of secretory proteins by omitting IPTG to BL21(DE3) cells cultured in LB medium

It has been shown that saturating the Sec-translocon capacity can also hamper the production of secretory proteins in the periplasm [26]. Therefore, we decided to explore the effect of omitting IPTG from BL21(DE3) cultures on the production of secretory Super folder Green Fluorescent Protein (SfGFP), which has a modified DsbA signal sequence at its N-terminus: DsbA*sfGFP [26].

**Fig. 6:**
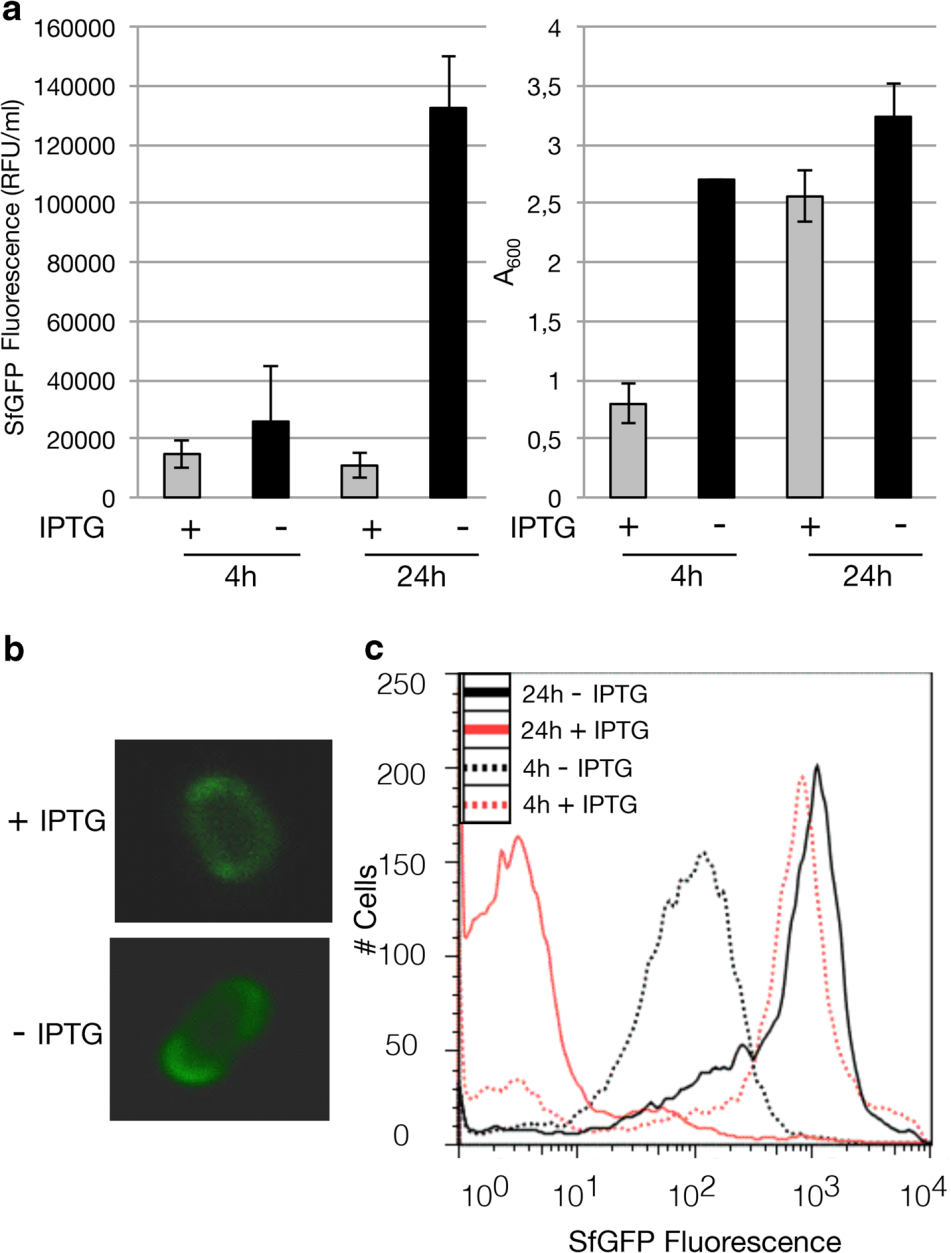
Production of secretory SfGFP in BL21(DE3) in the presence and absence of IPTG. BL21(DE3) cells harboring a pET-based dsbA*sfgfp expression vector were cultured in LB medium at 30 °C in the absence and presence of IPTG (final concentration 0.4 mM). **a** To assess SfGFP production levels, we monitored 4 and 24 h after the addition of IPTG fluorescence (relative fluorescence unit, RFU) per milliliter of culture. Biomass formation was monitored by measuring the A_600_. RFUs/ml per A_600_ are shown in Additional file 1: Figure S1. **b** The localization of secretory SfGFP in BL21(DE3) cells cultured in the absence and presence of IPTG was monitored directly in whole cells using fluorescence microscopy. **c** The production of secretory SfGFP per cell was monitored using flow cytometry. Traces of cells cultured in the prescence of IPTG are in *red* and traces of cells cultured in the absence of IPTG are in *black*. Cells harvested after 4 h are represented by *dotted lines* and cells harvested after 24 h are represented by *solid lines*

The fluorescence intensities per ml of culture of un-induced BL21(DE3) cells harbouring pETdsbA*sfgfp were significantly higher than the ones of IPTG induced cultures; after 24 h, these values were approximately ten fold higher (Fig. 6a). The amount of biomass formed was negatively affected by IPTG (Fig. 6a). Also the fluorescence intensities per ml of culture obtained for un-induced BL21(DE3) cultures were higher than the ones obtained for C41(DE3) and C43(DE3) based cultures (results not shown). Analysis of BL21(DE3) cells producing SfGFP cultured in the absence and presence of IPTG using fluorescence microscopy resulted in green fluorescent halos which indicates that the SfGFP was efficiently translocated across the membrane to the periplasm (Fig. 6b) [26]. Next, using flow cytometry we showed that cultures producing secretory SfGFP in the absence of IPTG consisted of a homogenous population of cells, both after 4 and 24 h, and that the fluorescence per cell increased over time (Fig. 6c). When cells were cultured for 4 h in the presence of IPTG the fluorescence per cell was significantly higher than in the absence of IPTG, but after 24 h the fluorescence per cell had decreased dramatically and the number of non-producing cells had increased (Fig. 6c). The highest GFP fluorescence intensities, both per ml of culture and per cell, were obtained after 24 h in the absence of IPTG (Fig. 6a, c).

**Fig. 7:**
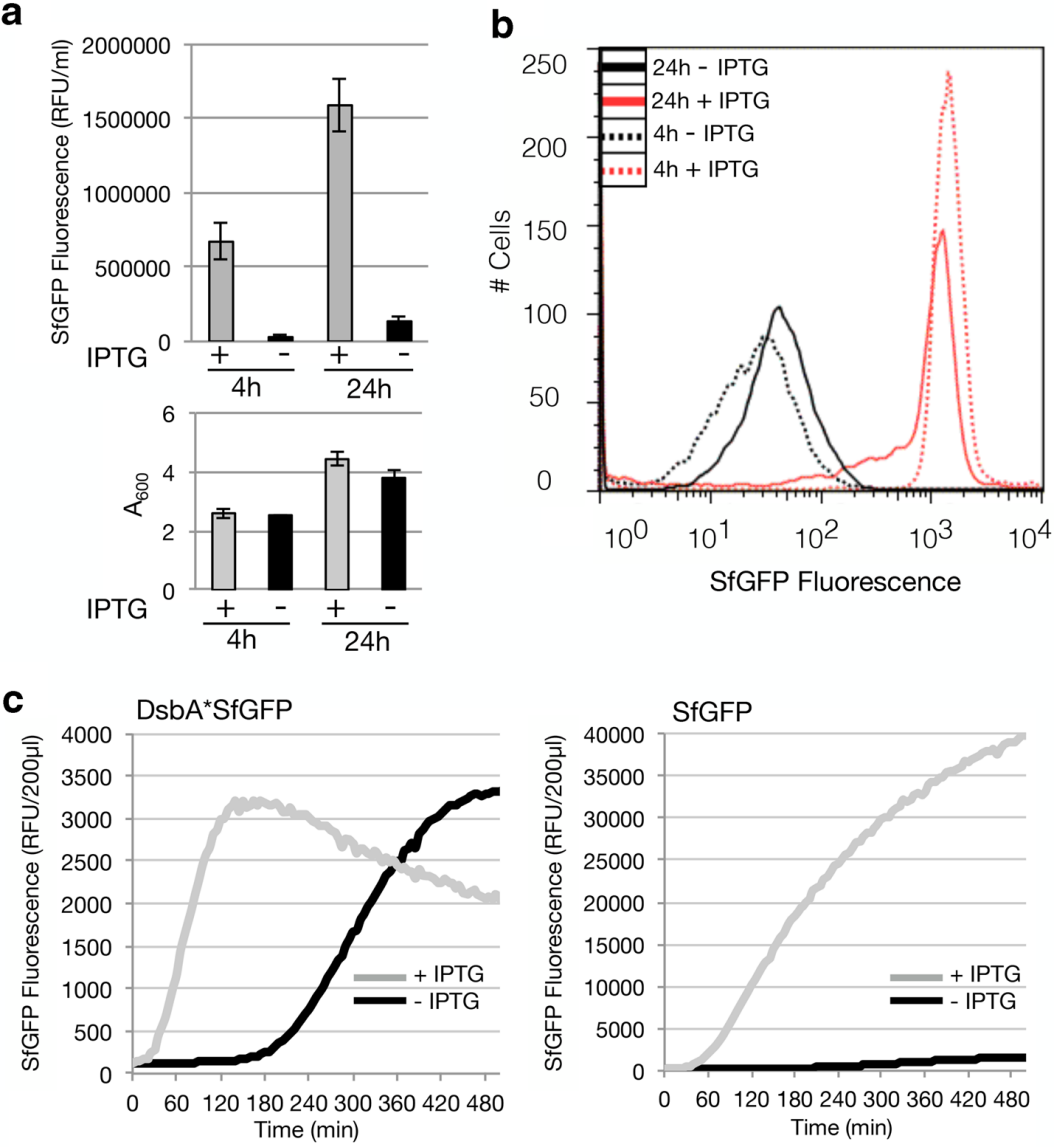
Production of cytoplasmic SfGFP in BL21(DE3) in the presence and absence of IPTG. BL21(DE3) cells harboring a pET-based *sfgfp* expression vector were cultured in LB medium at 30 °C in the absence and presence of IPTG (final concentration 0.4 mM IPTG). **a** To assess SfGFP production levels, we monitored 4 and 24 h after the addition of IPTG fluorescence (relative fluorescence unit, RFU) per milliliter of culture. Biomass formation was monitored by measuring the A_600_. RFUs/ml per A_600_ are shown in Additional file 1: Figure S1. **b** The production of SfGFP per cell was monitored using flow cytometry. Traces of cells cultured in the presence of IPTG are *red* and traces of cells cultured in the absence of IPTG are *black*. Cells harvested after 4 h are represented by dotted lines and cells harvested after 24 h are represented by* solid lines*. The time point at which IPTG was added to the +IPTG cultures was taken as 0 h. **c** The production of SfGFP in the periplasm (*left panel*) and in the cytoplasm (*right panel*) was monitored on-line by measuring GFP fluorescence every 5 min in cells cultured in the presence and absence of IPTG in a 96-well plate in a spectrofluorometer. Traces representing cells cultured in the presence of IPTG are in *grey* and traces representing cells cultured in the absence of IPTG are in *black*

As a control, we also produced SfGFP without a signal sequence in BL21(DE3) in the presence and absence of IPTG. In contrast to secretory SfGFP, cytoplasmic SfGFP was more efficiently produced in the presence of IPTG than in its absence (Fig. 7a). Using flow cytometry experiments showed that addition of IPTG had hardly any negative effect on the amount of SfGFP produced per cell (Fig. 7b), which indicates that the production of SfGFP is indeed not toxic.

We further explored the differences between secretory SfGFP and cytoplasmic SfGFP production by measuring in real-time the accumulation of fluorescence in BL21(DE3)-based cultures, in the presence and absence of IPTG (Fig. 7c). When producing secretory SfGFP in the presence of IPTG, initially fluorescence accumulates rapidly and over time the levels decline. This is most likely due to a negative effect on growth/accumulation of non-producing cells. In contrast, when producing secretory SfGFP in cells in the absence of IPTG, fluorescence accumulates slowly but steadily and at some point exceeds the fluorescence accumulated in cells cultured in the presence of IPTG. In BL21(DE3)-based cultures producing cytoplasmic SfGFP in the presence of IPTG, fluorescence accumulates rapidly and steadily whereas in the absence of IPTG, fluorescence accumulates steadily but only slowly. This indicates that production of SfGFP is not toxic *per se*, but that translocation of the protein across the cytoplasmic membrane is the critical point. For two more secretory proteins we showed that they were produced more efficiently in BL21(DE3) cells cultured in the absence of IPTG than in the presence of IPTG and that produced proteins were suitable for further experimentation (Additional file 1: Figures S3, S4).

Taken together, omitting IPTG from BL21(DE3) cells cultured in LB medium leads to more efficient production of secretory proteins since the Sec-translocon capacity is not/hardly affected.

## Concluding remarks

We have shown that omitting IPTG from BL21(DE3) cells cultured in LB medium provides in many cases an effective, competitive and convenient alternative for the production of membrane proteins in the cytoplasmic membrane and secretory proteins in the periplasm. Therefore, we recommend that this condition is incorporated in membrane- and secretory protein production screens.

## Methods

### Strains, plasmids and culture conditions

For protein production experiments the *E. coli* strains BL21(DE3), C41(DE3) and C43(DE3) were used [4, 16]. All genes, but one, encoding the target proteins used in this study were expressed from a pET28a+ derived vector as described before [22]. The one exception is described in Additional file 1: Figure S4. All membrane protein targets were produced as C-terminal GFP-His_8_ fusions as described before [27]. Cells were grown aerobically at 30 °C and 200 rpm, in Lysogeny broth (LB) medium (Difco) supplemented with 50 µg/ml kanamycin. At an A_600_ of ~0.4 target gene expression was induced by adding 0.4 mM IPTG. Growth was monitored by measuring the A_600_ with a UV-1601 spectrophotometer (Shimadzu). For online GFP fluorescence measurements 200 µl of the induced (or not induced) cultures were transferred at an A_600_ of ~0.4 to a 96 well plate and fluorescence was automatically detected every 5 min. The 96 well plate was shaken every 30 s [13].

### Whole cell fluorescence measurements and flow cytometry

Production of membrane protein GFP fusions and secretory SfGFP were monitored using whole-cell fluorescence as described before [22]. Standard deviations are based on a minimum of three biologically independent experiments. GFP fluorescence was analyzed on a single cell level by flow cytometry using a FACSCalibur instrument (BD Biosciences) as described before [12]. FM4-64 membrane staining was used to discriminate between cells and background signal. The FlowJo software (Treestar) was used for raw data analysis/processing.

### SDS-PAGE, in-gel fluorescence and immuno-blotting

Whole cell lysates (0.05 A_600_ units) were analyzed by standard SDS-PAGE using 12 % polyacrylamide gels followed by either in-gel fluorescence or immuno-blotting as described before [22, 28]. His-tagged target membrane proteins were detected using an HRP-conjugated α-His antibody (ThermoFisher) recognizing the C-terminal His-tag. IbpB levels were monitored using antisera from our sera collection, followed by incubation with a secondary HRP-conjugated goat-α-rabbit antibody (Bio-Rad). Proteins were visualized using the ECL-system (GE Healthcare) according to the instructions of the manufacturer and a Fuji LAS-1000 charge coupled device (CCD) camera.

### Fluorescence microscopy

Prior to microscopy, cells were fixed using cross-linking reagents. Cells corresponding to 1 A_600_ unit were harvested (4000×*g*, 2 min) and resuspended in 1 ml phosphate buffered saline (PBS) pH 7.4. Subsequently, 1 ml fixing solution (5.6 % Formaldehyde, 0.08 % Glutaraldehyde in PBS) was added and cells were incubated for 15 min at room temperature. Subsequently, cells were washed three times with PBS and resuspended in 100 µl PBS. 1 µl of the cell suspension was mounted on a glass slide. Fluorescence images of cells expressing secretory SfGFP were obtained using a light scanning microscope (LSM 700) set-up (Zeiss). The resulting images were processed with the AxioVision 4.5 software (Zeiss).

### Fluorescence-detection size-exclusion chromatography

1 L cultures of BL21(DE3) cells producing the YidC-GFP-fusion were used as starting material for the isolation of membranes. All steps involved in the isolation of the membrane fraction were carried out either on ice or at 4 °C. Isolated cells were broken with five passes through an Emulsiflex-C3 (Avestin), at 10,000–15,000 psi. The lysate was cleared of unbroken cells by centrifugation (8000×*g*, 3 × 20 min, 4 °C). Membranes were isolated by centrifugation for 1 h at 45,000×*g* and resuspended in 10 mL PBS buffer. An amount corresponding to 5000 RFU were solubilized by incubation in 1 ml PBS containing 1 % DDM for 1 h at 4 °C, with continuous stirring. Non-solubilized membranes were removed by ultracentrifugation at 120,000×*g* for 45 min. 100 µl of solubilized material was loaded onto a Superose 6 column (10/30, GE-healthcare) pre-equilibrated with 20 mM Tris–HCl pH 7.5, 150 mM NaCl, 0.03 % (w/v) DDM at a flow rate of 0.3 mL/min. GFP fluorescence was monitored (emission wavelength of 512 nm and excitation wavelength of 488 nm) using an inline-detector Shimadzu HPLC system (Shimadzu Corporation).

### Isolation of GltP-GFP and GltP activity assay

1 L cultures of BL21(DE3) cells producing the GltP-GFP-fusion were used as starting material for the isolation of membranes. Membranes were isolated as described under ‘Fluorescence-detection size-exclusion chromatography’. The IMAC-based purification of the GltP–GFP fusion and the GltP activity assay were performed as described previously [29].

## Additional file

Additional file 1. In the Supplemental Material Section properties of the membrane proteins used in this study are provided and results from the membrane and secretory protein production experiments are presented.

## Abbreviations

T7 RNAP: T7 RNA polymerase
LB: lysogeny broth
IPTG: isopropyl-β-d-thiogalactoside
GFP: green fluorescent protein
SfGFP: super folder green fluorescent protein
DsbA: dithiol-disulfide oxidoreductase A
SDS-PAGE: sodium dodecyl sulfate polyacrylamide gel electrophoresis
CCD: charge coupled device
IbpB: inclusion body protein B
IMAC: Immobilized-Metal Affinity Chromatography
DDM: n-dodecyl β-d-maltoside
FSEC: fluorescence-detection size-exclusion chromatography
PBS: phosphate buffered saline
scFv: single-chain variable antibody fragment
TM: transmembrane domains
OCC: octaheme *c*-type cytochrome
OmpA: outer membrane protein A
TEV protease: tobacco etch virus protease
