# Supplementary material for: High-level production of membrane proteins in E. coli BL21(DE3) by omitting the inducer IPTG
Source: Microb Cell Fact. 2015 Sep 16;14:142. doi: 10.1186/s12934-015-0328-z (PMC4574001; doi:10.1186/s12934-015-0328-z)
Supplement: Additional file 1. — In the Supplemental Material Section properties of the membrane proteins used in this study are provided and results from the membrane and secretory protein production experiments are presented. [file 12934_2015_328_MOESM1_ESM.pdf]

Additional file 1. Table S1 and Figures S1-4 of Zhang *et al.* 'High-level production of membrane proteins in *E. coli* BL21(DE3) by omitting the inducer IPTG'

Table S1. Properties of the membrane proteins used for the screen in Figure 5. The information listed: names of the membrane proteins used, organism, function, number of amino acids, number of transmembrane domains (TMs)/predicted TMs and topology of the membrane protein ($N_{in/out}$ and $C_{in/out}$) [1].

Protein	Organism	Function	Length (amino acids)	Number of TMs/ predicted TMs	N_{in}/N_{out} & C_{in}/C_{out} topology
**TehA**	*Haemophilus Influenza*	Tellurite resistance protein/Ethidium efflux transporter/ proflavin transporter	330	10	N_{in}/C_{in}
**YijD**	*Escherichia coli*	Putative inner membrane protein: function unknown	119	4	N_{in}/C_{in}
**PheP**	*Escherichia coli*	Phenylalanine transporter	458	12	N_{in}/C_{in}
**YgfU**	*Escherichia coli*	Urate symport protein	482	10	N_{in}/C_{in}
**YqcE**	*Escherichia coli*	Putative inner membrane protein: function unknown	425	12	N_{in}/C_{in}
**YfbF**	*Escherichia coli*	Undecaprenyl phosphate-L-Ara4FN transferase/polymyxin resistance protein	322	2	N_{in}/C_{in}
**GltP**	*Escherichia coli*	Glutamate aspartate symport protein	437	8	N_{in}/C_{in}
**YidC**	*Escherichia coli*	Inner-membrane protein insertion factor	548	6	N_{in}/C_{in}

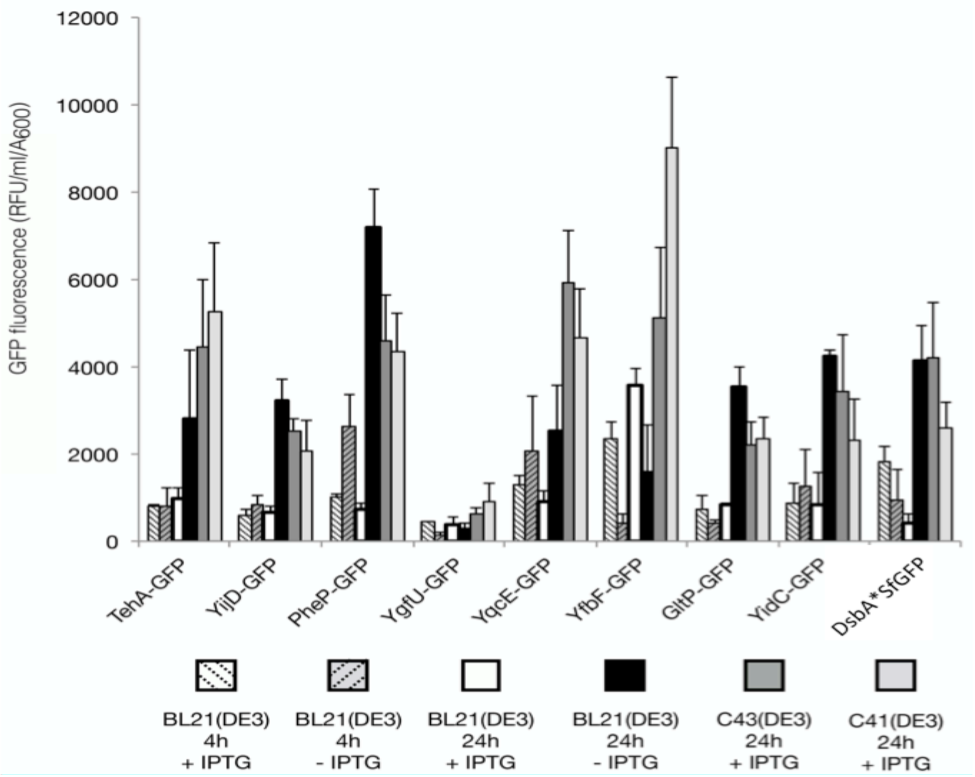

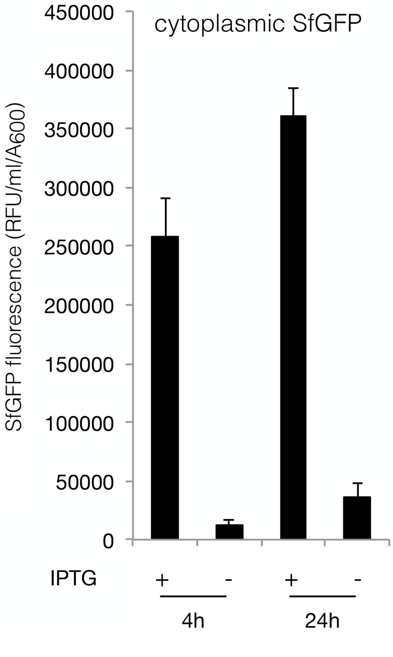

Figure S1. Protein production normalised to cell density. Top panel. The fluorescence (RFU/ml) and A_{600} data in Figures 2a, 5 and 6a are here presented as RFU/ml/A_{600}. For graphical reasons, fluorescence values of DsbA*SfGFP were divided by 10. Bottom panel. The fluorescence (RFU/ml) and A_{600} data in Figure 7a are here presented as RFU/ml/A_{600}.

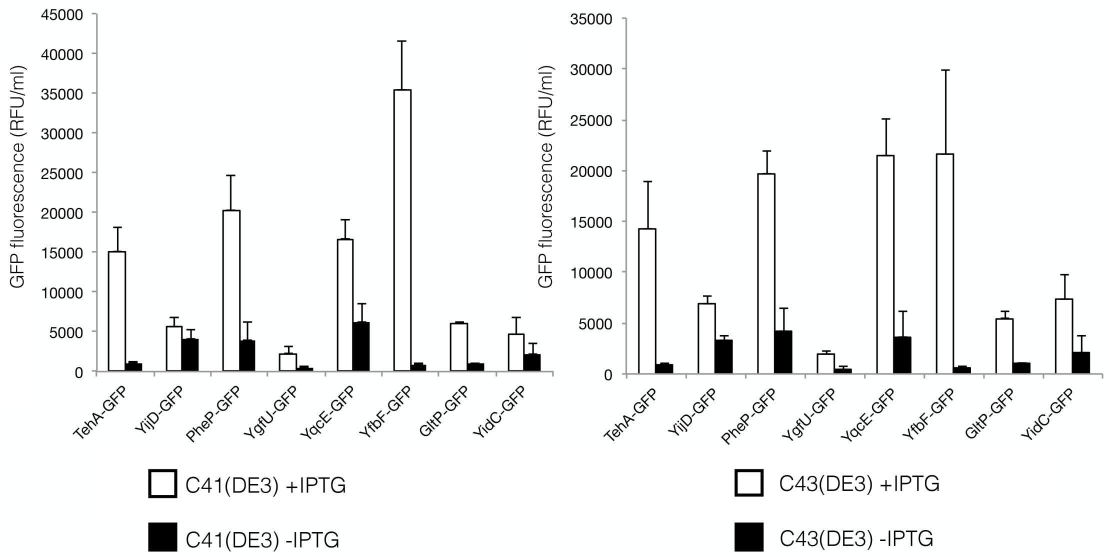

Figure S2. Screening the production of membrane proteins in C41(DE3) and C43(DE3) in the absence of IPTG. The production of the set of membrane protein GFP-fusions (See Additional material, Table S1) was not only, as shown in Figure 5, assessed in C41(DE3) and C43(DE3) cells cultured in the presence of IPTG, but also in C41(DE3) and C43(DE3) cells cultured in the absence of IPTG. Fluorescence per ml of culture was monitored 24 hours after IPTG had been added. For the sake of clarity the data obtained for C41(DE3) and C43(DE3) cells cultured in the presence of IPTG presented in Figure 5 in the main text are also included in this figure. Data from C41(DE3) experiments are presented in the left panel and data from C43(DE3) experiments are presented in the right panel.

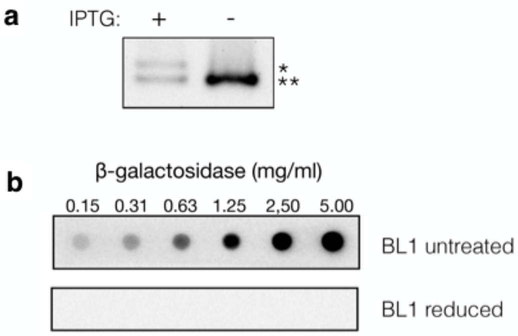

Figure S3. Production of the single-chain variable fragment (scFv) BL1 in BL21(DE3) in the presence and absence of IPTG. The scFv BL1 N-terminally fused to a recently described modified DsbA signal sequence was produced in BL21(DE3) cells cultured in LB medium in the presence and absence of IPTG as described in 'Methods' [2]. Notably, BL1 contains a C-terminal His-tag, facilitating its detection by means of immuno-blotting. **a** BL1 production in BL21(DE3)pET*dsbA*bl1* cells was analyzed 4 hours after the addition of IPTG to the + IPTG culture by means of immuno-blotting. For immuno-blotting equal amounts of cells were loaded per lane and an HRP-conjugated α-His antibody (ThermoFisher) was used for detection (see Methods). * = precursor form of the protein (DsbABL1; cytoplasmically localized) and ** = processed form of the protein (BL1; periplasmically localized). **b** Top panel. The proper folding of BL1 produced in BL21(DE3) cells cultured in the absence of IPTG was assayed by the recognition of its substrate, *E. coli* β-galactosidase, using a dot-blot assay and whole cell lysate as decribed before [2]. Bottom panel. As a control the whole lysate was treated with the reductant β-mercaptoethanol as describe before [2].

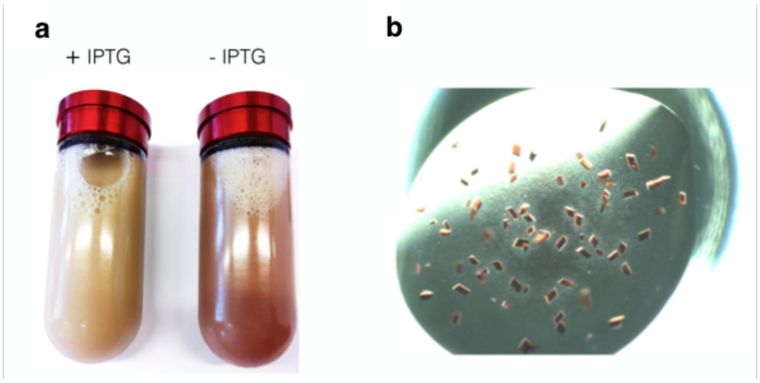

Figure S4. Production of a bacterial octaheme *c*-type cytochrome in BL21(DE3) in the presence and absence of IPTG. The bacterial octaheme *c*-type cytochrome (OCC) was fused to its N-terminus to the *E. coli* OmpA signal sequence and to its C-terminus to a single StrepTag II (WSHPQFEK) and a TEV protease cleavage site. The gene encoding the OCC was expressed from a modified pET22a vector and it was codon optimized by GeneArt (Invitrogen). For the production of the OCC, *E. coli* BL21(DE3) containing pEC86_*ccmABCDEFGH* and pET22*occ* were cultivated overnight in one litre of LB medium containing 100 μg/ml ampicillin and 30 μg/ml chloramphenicol at 30°C as described in 'Methods'. pEC86_*ccmABCDEFGH* is required for the maturation of in the periplasm secreted OCC [3, 4]. Incorporation of the heme moiety during maturation of the OCC in the periplasm gives the protein a red color. **a** Isolated BL21(DE3) pEC86_*ccmABCDEFGH*pET22*occ* cells cultured in the presence and absence of IPTG were broken using an Emulsiflex; the left tube contains a lysate prepared from cells cultured in the presence of IPTG and the right tube contains a lysate prepared from cells cultured in the absence of IPTG. Culturing cells in the absence of IPTG leads to 10-20% more biomass formation and lysates prepared of these cells are considerably more red colored than lysates prepared from cells cultured in the presence of IPTG. This indicates that omitting IPTG leads to enhanced production of mature OCC. **b** For purification of the mature OCC, the lysate derived of cells cultured in the absence of IPTG was applied to a Streptactin column (IBA), followed by purification by a Superdex 200 XK 16/60 (GE Healthcare) size exclusion chromatography. The final yield was 0.21 mg of OCC per liter of culture. Isolated material was subsequently crystallized (see picture) and the crystals diffracted to 2.3 Å.

References

1. Daley DO, Rapp M, Granseth E, Melen K, Drew D, von Heijne G: **Global topology analysis of the *Escherichia coli* inner membrane proteome**. *Science* 2005, **308**(5726):1321-1323.
2. Schlegel S, Rujas E, Ytterberg AJ, Zubarev RA, Luirink J, de Gier JW: **Optimizing heterologous protein production in the periplasm of *E. coli* by regulating gene expression levels**. *Microb Cell Fact* 2013, **12**:24.
3. Thony-Meyer L, Fischer F, Kunzler P, Ritz D, Hennecke H: ***Escherichia coli* genes required for cytochrome *c* maturation**. *J Bacteriol* 1995, **177**(15):4321-4326.
4. Arslan E, Schulz H, Zufferey R, Kunzler P, Thony-Meyer L: **Overproduction of the *Bradyrhizobium japonicum* *c*-type cytochrome subunits of the cbb_3 oxidase in *Escherichia coli***. *Biochem Biophys Res Commun* 1998, **251**(3):744-747.
